# Paradoxical Psoriasis and Worsening Spondylitis Due to Secukinumab in a Patient With Ankylosing Spondylitis: A Case Report and Literature Review

**DOI:** 10.7759/cureus.50726

**Published:** 2023-12-18

**Authors:** Khalid A Alnaqbi, Jawaher Al Zeyoudi, Fahad Fazal, Omar M Alhaj, Imad Jassim, Fatima A Albreiki

**Affiliations:** 1 Internal Medicine, Tawam Hospital and College of Medicine & Health Sciences, United Arab Emirates University, Al Ain, ARE; 2 Internal Medicine/Rheumatology, Tawam Hospital, Al Ain, ARE; 3 Academic Affairs, Sheikh Shakhbout Medical City, Abu Dhabi, ARE; 4 Rheumatology, Mediclinic, Abu Dhabi, ARE; 5 Academic Affairs, Tawam Hospital, Al Ain, ARE; 6 Rheumatology, Tawam Hospital, Al Ain, ARE; 7 Dermatology, Tawam Hospital, Al Ain, ARE

**Keywords:** upadacitinib, jak inhibitor, paradoxical arthritis, paradoxical reaction, secukinumab, il-17 inhibitor therapy, paradoxical psoriasis, psoriasis, axial spondyloarthritis, ankylosing spondylitis

## Abstract

Axial spondyloarthritis (axSpA) is an autoimmune disease primarily affecting the axial skeleton, with associated extra-musculoskeletal manifestations. Treatment strategies targeting cytokines tumor necrosis factor-alpha (TNF-α) and interleukin 17 (IL-17) have proven effective. However, paradoxical reactions, including paradoxical psoriasis and arthritis, have been reported in axSpA patients receiving TNF-α inhibitors. IL-17 inhibitors have been used as an alternative treatment option, but paradoxical reactions have also been rarely observed. This case report presents a 45-year-old man with axSpA who responded to infliximab for six years before discontinuing it due to secondary failure. After the washout period of infliximab, he was started on secukinumab but developed paradoxical psoriasis and worsening of inflammatory back pain after receiving the second loading dose which necessitated replacing it with upadacitinib. Complete resolution of paradoxical psoriasis and significant improvement in his back pain after three months ensued. This case contributes to understanding the complex dynamics in treating axSpA and managing paradoxical reactions.

## Introduction

Axial spondyloarthritis (axSpA) is a chronic idiopathic inflammatory disease that primarily affects the axial skeleton and is classified into non-radiographic axSpA and radiographic axSpA (ankylosing spondylitis). Patients may also develop enthesitis, peripheral arthritis, and extra-musculoskeletal manifestations such as uveitis, psoriasis, and inflammatory bowel disease (IBD). This condition can cause significant disability due to ankylosis and loss of spinal mobility and work absenteeism [1]. The 2022 Assessment of Spondyloarthritis International Society (ASAS)-European Alliance of Associations for Rheumatology (EULAR) recommendations for treating axSpA include non-pharmacological and pharmacological interventions, including interleukin 17 (IL-17) inhibitors, secukinumab, and ixekizumab [2].

With the wide use of biologics in the management of autoimmune diseases, paradoxical reactions (PRs), including paradoxical psoriasis (PP) and paradoxical arthritis, have been reported in the literature, particularly with tumor necrosis factor (TNF) inhibitors [3]. PRs are unexpected and contrary responses to a drug where the intended therapeutic effect is not achieved and new or worsening symptoms occur. These reactions are considered paradoxical because they oppose the expected outcome based on the drug’s mechanism of action or established therapeutic indications [4].

Our case report aims to present a unique case of PP and worsening inflammatory back pain due to IL-17 inhibitor, secukinumab, in a patient with axSpA. We also describe a review of the literature and summarize the clinical characteristics, management approach, and treatment outcomes of the published cases.

## Case presentation

**Figure 1:**
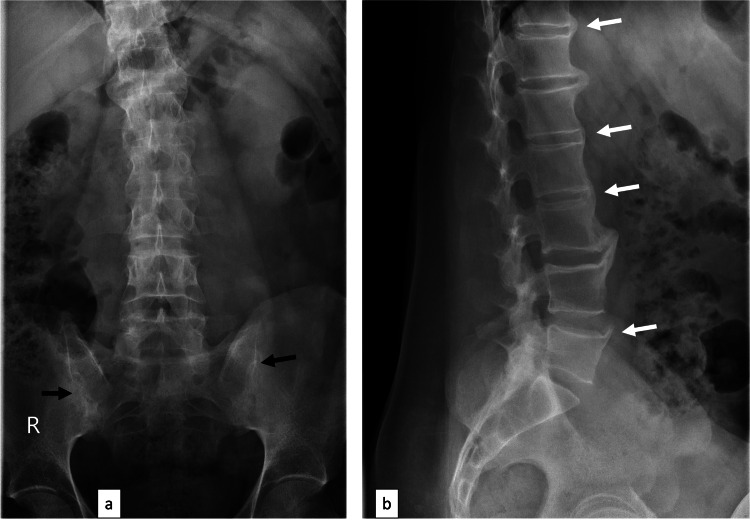
X-rays of the lumbosacral spine (a) anteroposterior and (b) lateral view showing sacroiliitis grade 3 bilaterally (black arrows) and lumbar syndesmophytes (white arrows) confirming the diagnosis of ankylosing spondylitis.

A 45-year-old man of Egyptian descent presented with a history of obesity, hypertension, smoking, hyperlipidemia, and a previously treated left deep venous thrombosis (provoked by a traumatic fracture of the left leg and complicated by pulmonary embolism in 2017). He had a long-standing history of typical inflammatory back pain since the age of 18. At the end of 2016, he saw a rheumatologist when X-rays of the lumbosacral spine and sacroiliac joints (SIJs) showed lumbar syndesmophytes and bilateral grade 3 sacroiliitis (Figure 1).

He was diagnosed with ankylosing spondylitis (AS) and fulfilled the modified New York criteria for AS. He had no personal or family history of peripheral arthritis, dactylitis, uveitis, psoriasis, or IBD. Due to the failure of many non-steroidal anti-inflammatory drugs, he was started on infliximab (Remicade) 5 mg/kg (500 mg) in April 2016. Methotrexate 10 mg orally weekly was added from 2016 to November 2022 as a concomitant therapy with infliximab (500 mg every eight weeks) in an attempt to reduce the formation of human anti-chimeric antibodies. His back pain improved.

On October 24th, 2022, he presented to our rheumatology clinic for a second opinion. He complained of recurrent severe lower back pain that affected his daily living and work. He reported a two-hour morning stiffness. He denied any trauma or infection. His other home medications included amlodipine-perindopril, rosuvastatin, and pantoprazole. On physical examination, his body mass index was 33.5 kg/m^2^, his blood pressure was 158/100 mmHg, and his heart rate was 80 beats/minute. The cervical range of motion was normal, while the occiput-to-wall distance was wide (7 cm), and chest expansion was normal (7 cm). The modified Schober test indicated restricted lumbar flexion at 1 cm, and lumbar lateral flexion was restricted bilaterally (7 cm). No joint swelling was observed. His blood tests revealed elevated C-reactive protein (CRP) of 12.4 mg/L (normal range: ≤5), negative anti-nuclear antibody, negative rheumatoid factor, negative hepatitis B and C serology, and a positive human leukocyte antigen B27.

On November 21st, 2022, due to persistent back pain, he returned to his primary rheumatologist and received his last infliximab infusion. His back pain did not improve afterward.

On January 4th, 2023, he was taking a muscle relaxant and etoricoxib. His Bath Ankylosing Spondylitis Disease Activity Index (BASDAI) score was 5/10, and his Ankylosing Spondylitis Disease Activity Score (ASDAS) score was 2.3, with both scores indicating high disease activity. His CRP level was elevated at 15.0 mg/L.

**Figure 2:**
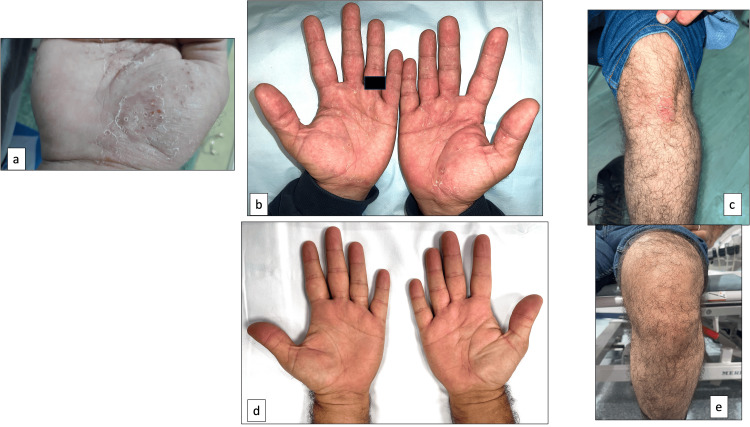
Image (a) shows pustular psoriasis on the palm on January 20th, 2023, after receiving the second loading dose of secukinumab. On March 8th, 2023, image (b) shows plaque psoriasis on the left knee, and image (c) shows dried-up pustules on the palms, leaving behind fringes of desquamation after one week of receiving the fifth loading dose of secukinumab. Images (d and c) show complete resolution of psoriasis on the hands and left knee after three months of upadacitinib.

The 2022 ASAS-EULAR recommendations are that after the failure of biological disease-modifying anti-rheumatic drugs (DMARDs) (in our case TNF inhibitor infliximab) in managing axSpA, a switch to another biologic DMARD should be considered (TNF inhibitor or IL-17) or JAK inhibitor with no specific drug preference [2]. On January 11th, 2023, he started taking his first loading dose of secukinumab 150 mg subcutaneously. After receiving two doses of secukinumab, he complained of a new pruritic rash on the palms and soles of his feet. On January 20th, 2023, he saw our dermatologist who diagnosed him clinically with palmoplantar pustular psoriasis (Figure 2, Panel a), which was treated with emollient and betamethasone dipropionate topical 0.064%-calcipotriol 0.005% ointment.

On February 8th, 2023, he took the fifth dose of secukinumab, and after a week, he complained of worsening back pain. On March 8th, 2023, his spondylitis disease activity was high (very high ASDAS 5.0, high BASDAI score 8.6, high CRP 12.9 mg/L). His palmoplantar pustular psoriasis worsened significantly, and new plaque psoriasis on his right elbow and left knee developed (Figure 2, Panels b, c), despite using topical therapy and taking secukinumab. Again, he reported no triggering factors for the rash, such as stress, trauma, new drugs (except secukinumab), or infections. He was prescribed naproxen 500 mg twice daily for his back pain. During a multidisciplinary meeting between rheumatology and dermatology, it was agreed to continue topical therapy and replace secukinumab with JAK inhibitor, upadacitinib 15 mg daily, starting on March 10th, 2023. The goal was to treat both conditions, axSpA and PP. He also stopped smoking.

After one month of starting upadacitinib, the patient reported significant improvement in his back pain and significant resolution of his psoriasis. He became independent in his daily living. His CRP decreased to 7.3 mg/L. He was referred to physiotherapy to optimize his back mobility and was continued on naproxen. His psoriasis completely resolved after three months of taking upadacitinib (Figure 2, Panels d, e). He sustained improvement in back pain without skin symptoms for nine months.

## Discussion

PRs can complicate treatment plans, leading to suboptimal or inadequate therapeutic responses to primary autoimmune disease. PRs may also result in the discontinuation or alteration of medications, necessitating further adjustments and potentially limiting treatment options [5].

A multi-center Turkish study recruited 8,192 patients, including 2,867 with RA and 5,316 with SpA. Out of 136 patients (1.66%) who experienced PRs, most were due to TNF inhibitors, with only one (0.7%) patient on secukinumab. Among those with PRs, 60% had them from their first biologic drug. The median time between PR onset and starting biologic treatment was 12 months. PP types included pustular (60.3%) and palmoplantar (30.9%). Switching to another biological drug was common among patients with PPs (73.2%). Progression of PPs occurred in only 5.2% of patients who switched to another TNF inhibitor. Interestingly, the study also noted a significantly higher number of smokers in patients with SpA [6].

A literature review was performed to search for English-language articles using PubMed, Web of Science, and Google Scholar databases. Search terms included “secukinumab,” “paradox,” “psoriasis,” “psoriatic,” “ankylosing spondylitis,” and “spondyloarthritis.” No time limits were applied. In addition, the available references were manually searched and reviewed to capture all potential cases.

We identified 18 cases with PP after secukinumab. Only five cases had spondyloarthritis. Only two out of the 18 cases were published in rheumatology journals, while the rest were published in dermatology journals. Table 1 shows a detailed description of the cases, while Table 2 summarizes the findings.

**Table 1:** Published cases of paradoxical psoriasis related to secukinumab. *: The period of follow-up was not stated. ^†^: Skin biopsy was not performed. F: female; M: male; NA: data not available; PsO: psoriasis; IFX: infliximab; SEC: secukinumab

Case number	Reference (year)	Primary disease	Age, sex	Current smoking history	SEC dose	Approximate duration of SEC use before paradoxical PsO	Previous history of biologic use	Paradoxical reaction(s)	Treatment	Outcome
1	Su et al. (2017) [7]	Psoriatic arthritis (with plaque PsO)	45, M	NA	300 mg (fourth loading dose)	4 weeks	Yes (etanercept, adalimumab, ustekinumab, IFX)	A flare of PsO.^†^ Severe bilateral uveitis and worsening axial symptoms	Discontinuation of SEC. Restarted IFX	Uveitis and axial pain subsided, but psoriasis did not*
2	Noell et al. (2017) [8]	Plaque PsO	53, F	NA	300 mg	Shortly after starting loading doses (duration not specified)	Yes (adalimumab)	New palmoplantar and inverse PsO^†^	Discontinuation of SEC. Prescribed IFX and topical steroids	Significant improvement*
3	Sladden et al. (2017) [9]	Plaque PsO (PsO vulgaris)	61, F	NA	NA	12 weeks	Yes (adalimumab)	New severe nail PsO^†^	Discontinuation of SEC after 6 months. Initiation of ustekinumab 45 mg after 2 months of stopping SEC with topical methotrexate	Significant improvement after 3 months
4	Hoshina et al. (2018) [10]	Psoriatic arthritis (with plaque PsO)	43, F	NA	300 mg	4 weeks	Yes (IFX)	Flare of PsO with visual deterioration (skin biopsy was performed)	Discontinuation of SEC. Restarted IFX but failed. Prescribed brodalumab but failed Prescribed cyclosporine 200 mg/day	Gradual improvement of psoriasis and vision was regained*
5	Prussick et al. (2018) [11] case 1	Plaque PsO	53, F	NA	300 mg	4 weeks	Yes (adalimumab)	New palmoplantar and inverse PsO^†^	Discontinuation of SEC. Prescribed ustekinumab and topical steroids for 2 months but failed. Prescribed IFX	Mild improvement*
6	Prussick et al. (2018) [11] case 2	Inverse PsO	84, M	NA	300 mg	4 weeks	Yes (adalimumab)	Flare of PsO and new palmoplantar PsO^†^	Discontinuation of SEC. Prescribed golimumab	Unknown
7	Dogra et al. (2019) [12]	Plaque PsO	22, M	NA	300 mg	38 weeks	No	Flare of PsO and new pustular PsO^†^	Discontinuation of SEC. Prescribed IFX	Complete resolution after 6 weeks
8	Currado et al. (2019) [13]	Ankylosing spondylitis	54, F	NA	150 mg	~40 weeks	Yes (adalimumab, etanercept, IFX)	New inverse PsO^†^	Discontinuation of SEC. Prescribed calcipotriol- betamethasone cream, and oral NSAIDs	Complete resolution after 1 month
9	Honma et al. (2019) [14]	Generalized pustular psoriasis	64, F	NA	300 mg	NA	No	New paradoxical palmoplantar pustulosis (skin biopsy proven)	SEC was continued. Prescribed topical steroids	Not worsening
10	Mössner et al. (2020) [15] Case 1	Psoriatic arthritis (with plaque PsO)	44, F	NA	300 mg	69.5 weeks	Yes (adalimumab)	New pustular palmoplantar PsO^†^	Topical therapy with potent corticosteroids. Discontinuation of SEC after 16 weeks of paradoxical PsO	Complete resolution after 4 weeks of stopping SEC
11	Mössner et al. (2020) [15] Case 2	Psoriatic arthritis (with plaque PsO)	45, F	Yes	150 mg	14 weeks	No	New pustular palmoplantar PsO^†^	Discontinuation of SEC. Topical steroids therapy. Prescribed ustekinumab	Significant improvement in skin lesions after 7 months
12	Abburuzzese et al. (2020) [16]	Psoriatic arthritis	63, F	NA	300 mg	26 weeks	Yes (adalimumab, etanercept, golimumab)	New pustular plantar PsO†	Cyclosporin 3 mg/kg was added to SEC	Complete resolution within 1 month
13	Durmaz et al (2020) [17]	Ankylosing spondylitis	47, F	NA	150mg	6 weeks (development of PsO coincided with the second course of SEC treatment)	No	New plaque PsO (clinically and confirmed by skin biopsy) after the second SEC dose	SEC was continued at the same dose. Topical calcipotriol plus betamethasone dipropionate were prescribed	Improved with residual post-inflammatory hyperpigmentation*
14	Penalba-Torres et al. (2022) Case 1 [18]	Hidradenitis suppurativa	36, F	Yes	300 mg	14 weeks	No	New palmoplantar pustulosis^†^	SEC continued for 9 months with topical clobetasol propionate and tacalcitol, then discontinued due to persistent and uncomfortable lesions	Complete resolution after 15 months on topical therapy alone
15	Penalba-Torres et al. (2022) Case 2 [18]	Spondyloarthropathy	54, F	Yes	150 mg	4 weeks	Yes (etanercept caused self-limited paradoxical psoriasis >10 years earlier)	New palmoplantar pustulosis^†^	Discontinuation of SEC at week 5 of SEC dosing	Significant improvement after 14 months with residual lesions, which are adequately controlled with topical treatment
16	El-Komy et al. (2022) [19]	Psoriatic arthritis (with plaque PsO)	46, F	NA	150 mg	Total 33 weeks of SEC intake (patient was on SEC for ~30 weeks, then stopped due to financial reasons, and was restarted with a flare of PsO after 3 weeks)	No	Flare of PsO	The dose was adjusted to 150 mg every 3 weeks, and acitretin 25 mg/day was added	Almost complete resolution except for the dorsum of hands. Acitretin was stopped 2 weeks after remission. Joint pain improved marginally
17	Urun et al. (2022) [20]	Plaque PsO	45, F	No	300mg	Week 11 of treatment	No	New palmoplantar pustulosis (clinically and confirmed by skin biopsy)	Discontinuation of SEC. Ustekinumab 45 mg combined with topical steroids was prescribed	Good response after 4 months
18	Anghel et al. (2023) [21]	Ankylosing spondylitis	55, F	NA	150 mg	After 26 weeks of treatment	No	New pustular palmar PsO (clinically and confirmed by skin biopsy)	Increased SEC to 300 mg/month, initiation of acitretin, topical mometasone, and narrow-band ultraviolet B phototherapy. Lesions worsened psoriasis after 2 months SEC was replaced with guselkumab without topical therapy	Partial remission after 3 months
19	Our case (2023)	Ankylosing spondylitis	45, M	Yes	150 mg	5 weeks	Yes (IFX)	New palmoplantar pustular psoriasis and plaque PsO^†^	Discontinuation of SEC after the 5th dose. Prescribed upadacitinib 15 mg daily and topical steroids, calcipotriol, were continued	Complete resolution after 3 months

**Table 2:** Summary of published paradoxical psoriasis cases secondary to secukinumab. JAK: janus kinase; SEC: secukinumab

Number of patients	19
Age (years)	Mean = 50.5. Median = 47
Sex, n (%)	females 15 (79%)
	males 4 (21%)
Primary disease	
Psoriasis only, n (%)	7 (36.8%)
Psoriatic arthritis, n (%)	6 (31.6%)
Spondyloarthritis, n (%)	5 (26.3%)
Hidradenitis suppurativa, n (%)	1 (5.3%)
Smoking history (n = 4/5)	80%
Previous history of biologic use, n (%)	11 (61.1%)
SEC dose and duration of intake	
Approximate duration of SEC use before paradoxical psoriasis occurred (weeks), n = 17	Mean = 19. Median = 14
Number of patients who received SEC 150 mg dose before paradoxical psoriasis occurred (n = 18), %	10 (55.6%)
Number of patients who received SEC 300 mg dose before paradoxical psoriasis occurred (n = 18), %	8 (47.1%)
Characteristics of paradoxical psoriasis (n = 19)	
Patients with only new (de novo) morphologic type of paradoxical psoriasis, n (%)	13 (68.4%)
Patients with paradoxical flare of established psoriasis, n (%)	3 (15.8%)
Patients with paradoxical psoriasis (flare of established psoriasis and new type of psoriasis), n (%)	3 (15.8%)
Performance of skin biopsy, n (%)	5 (26.3%)
Treatment for paradoxical psoriasis (n = 19)	
Continuing SEC with topical therapy	3 (15.8%)
Discontinuation of SEC and continuation of topical therapy	4 (21.0%)
Discontinuation of SEC and restarting previous biologic, n (%)	2 (10.5%)
Discontinuation of SEC + switching to ≥ one new biologic, n (%)	9 (47.4%)
Discontinuation of SEC + switching to JAK inhibitor, n (%)	1 (5.3%)
Outcome (n = 18)	
Resolution (partial or complete), n (%)	17 (94.4%)
No resolution, n (%)	1 (5.6%)

To summarize the published case reports, secukinumab can worsen axial symptoms and arthritis in cases with spondyloarthritis. PP is very rare with IL-17 inhibitor therapy, secukinumab, and occurs mostly in patients who have been exposed to TNF inhibitors. The mean duration of secukinumab use before PP is 19 weeks. Most patients had a new (de novo) morphologic type of PP. In most published cases, secukinumab was replaced with one or more new biologic DMARDs. Partial or complete resolution of symptoms is expected after approximately 10 months.

PP is relatively common in patients taking TNF blockers, particularly infliximab [22]. Interestingly, our patient’s presentation resembles a published case (number 15 on the table) where the patient with spondyloarthritis had self-limited PP induced by etanercept, which was discontinued 10 years before starting secukinumab [18]. However, our patient continued receiving infliximab for six years before its discontinuation due to secondary failure. It should be noted that the median half-life of infliximab ranges from 7.7 to 9.5 days, and the washout period is 29 days [23,24]. Therefore, it is highly unlikely that infliximab triggered the PP that started after 60 days of the last infusion, coinciding with the second loading dose of secukinumab. In our case, the PP started as new palmoplantar pustular psoriasis and progressed into plaque psoriasis affecting other areas of the skin, in addition to worsening spondylitis disease activity measures, including high CRP levels. Furthermore, this is the first case reporting PP related to secukinumab and resolved with JAK inhibitor, upadacitinib.

Our case highlights that PPs and arthritis are not only specific adverse events to TNF-α inhibitors but also possible adverse events of any biological drug interfering with the immune system. While the exact pathophysiology of induced PPs is not fully understood, several mechanisms have been reported. First, it is suggested that plasmacytoid dendritic cells may increase the production of interferon-gamma, leading to enhanced T-cell activity. Second, a decrease in T-regulatory cells and an imbalance in immune reactions may contribute to the development of PPs. Furthermore, the dysregulation of cytokines, particularly the IL-17 pathway, is considered crucial in the development of PRs induced by secukinumab [3]. Understanding the underlying mechanisms and risk factors associated with PRs can help guide treatment decisions.

Based on the literature review, management of PRs depends on their severity, primary underlying disease, and alternative treatment. The primary goals remain controlling the primary disease and treating the PR. Options to be considered for managing PRs include continuation of the biologic drug if the PR is mild, topical treatment and/or ultraviolet phototherapy for mild psoriasis, the addition of conventional synthetic DMARDs (e.g., glucocorticosteroids, methotrexate), and replacement of the offending drug with another class of immunomodulatory drugs such as TNF inhibitors, IL-17 inhibitors, IL12-23 inhibitor, or JAK inhibitors. Switching within the same class carries a risk of recurrence and, therefore, is best avoided [22,25]. Further research with a large number of patients may shed some light on the best management strategies for such cases.

## Conclusions

TNF-α inhibitors and IL-17 inhibitors have proven to be effective drugs for axSpA. While they are generally well tolerated, increasing cases associated with PRs have been reported, mostly associated with TNF inhibitors. PP and arthritis are very rare with secukinumab. Our axSpA case demonstrates developing de novo PP and worsening spondylitis after being treated with an IL-17 inhibitor, secukinumab. Switching to a different class of immunomodulators, such as JAK inhibitors, is a reasonable therapeutic option with success, as experienced by our patient. Close monitoring for PRs, prompt recognition, and appropriate management are essential in optimizing therapeutic outcomes and minimizing the burden of these rare adverse events in patients with axSpA. Further research is needed to understand the underlying mechanisms and develop strategies to manage these complications in axSpA patients effectively.
